# A scoping review of dental practitioners’ perspectives on perceived barriers and facilitators to preventive oral health care in general dental practice

**DOI:** 10.1186/s12903-024-04022-1

**Published:** 2024-02-17

**Authors:** H. Leggett, K. Vinall-Collier, J. Csikar, J. Owen, S. Edwebi, G. V.A Douglas

**Affiliations:** 1 York Trials Unit, The University of York, York, UK; 2 The School of Dentistry, The University of Leeds, Leeds, UK

**Keywords:** Oral health, Prevention, Dentistry, Review

## Abstract

**Background:**

Oral health has an important role in the general health and well-being of individuals. Dental teams are ideally placed to support patients in preventing ill-health. Understanding the barriers and facilitators to the adoption, promotion and facilitation of preventive advice and treatment is key to improving oral health services. The Theoretical Domains Framework (TDF) is a useful psychological framework to help identify individual, interpersonal and environmental issues which could be impacting clinicians’ ability to provide preventive advice and care. The aim of this review was to identify the perceived barriers and facilitators to preventive oral health care from the perspectives of the oral healthcare team within the general dental practice.

**Methods:**

A search strategy was developed, piloted, and run in: Medline via Ovid, PsycInfo, Web of Science, SCOPUS, EMBASE, Conference Proceedings Citation Index- Science, Cochrane Central Register of Controlled Trials and Cochrane Database of Systematic Reviews and followed PRISMA guidelines. Identified records were screened independently by two researchers. Data were coded using the Theoretical Domains Framework (TDF) and analysed using narrative data synthesis.

**Results:**

5610 papers were identified, and 19 included in this review. Thirteen papers focussed on dentists. Of the 106 items mapped onto the TDF, 48 were facilitators. The domains most frequently represented were, environmental context and resources, beliefs about consequences, social professional role and identity, skills, beliefs about capabilities and knowledge. Six studies focussed on dental hygienists. There were 47 items mapped onto the TDF, 18 were facilitators. The domains most frequently represented were environmental context and resources, social influences, beliefs about consequences and knowledge.

**Conclusions:**

The review identified that the delivery of preventive activities did not focus solely on the patient and dental professional interaction as many previous studies have highlighted. The review found that multiple factors influence whether prevention is delivered to patients. The largest barrier and facilitator for the dental professionals identified in this review was the environmental context and resources. Further research is needed to evaluate the effectiveness of interventions that aim to promote preventive oral health care in primary care settings to understand whether they address the barriers identified in this review.

**Supplementary Information:**

The online version contains supplementary material available at 10.1186/s12903-024-04022-1.

## Introduction

It is well recognised that oral health has an important role in the general health and well-being of individuals; with the risk factors for many general health conditions common to those that affect oral health, namely poor oral hygiene, smoking, alcohol use and a poor diet [1]. As such, it is of vital importance that all clinical teams make every contact count and support patients to make healthier choices [2]. There is currently a drive for greater emphasis on the prevention of ill-health and the reduction of inequalities of health through the delivery of advice, provision of support to change behaviour and application of evidence-informed actions [3]. However, it is not clear the extent to which this drive is being met or what is hindering or encouraging it within oral health care [4, 5].

The Adult Dental Health Survey 2009 [6], surveyed the general population about their experiences in attending the dentist. Responses showed that 78% of adults recalled being given advice at the dentist on cleaning their teeth or gums. This number had increased to nearly 90% (89.5%) in a more recent survey that asked adults who attended general dental practices in 2018 whether they recalled receiving prevention advice [7]. However, the Adult Dental Health Survey (2009) also found that 20% of patients were not satisfied with their dental visit. This was mainly related to the interaction between the dentist and the patient, highlighting the importance of the oral health conversation [8]. More positively, a survey from 2018 showed that 85.2% of patients had a positive experience of NHS dental services [9]. Those with a poor relationship with the dentist tend to rate their own oral health lower, leave longer intervals between visits to the dentist and are more likely to be extremely anxious about visiting a dentist [10]. Dental teams therefore have an important role in advising their patients about how they can make choices that improve and maintain their oral and general health. The publication of ‘Delivering better oral health – an evidence-based toolkit for prevention’, supported dental clinicians in adopting a new approach whereby all patients, were given preventive advice and offered preventive treatment [11]. This guidance lists the advice and actions that should be provided for all patients to maintain good oral health. However, it does not outline how the advice should be delivered. The delivery of prevention to patients is dependent upon dental professionals providing it; as such, their behaviour plays an important role in delivery.

The Theoretical Domains Framework (TDF) is a psychological framework that has been previously used to successfully identify important determinants of dental behaviours, in particular those of the dental health professional [12, 13]. The TDF [14] is a comprehensive list of the determinants of behaviour derived from 33 behaviour change theories. It identifies 14 key domains thought to influence behaviour, including knowledge, skills, motivation and goals, beliefs about capabilities, social influences, and behaviour regulation (See Table 1 for the full list and a description of each domain). Furthermore, it provides a valuable framework for assessing the psychological determinants of behaviour at all levels of influence (individual, interpersonal and environmental); thus, it provides an underlying scientific rigor and allows the mechanism of action within interventions to be studied.

**Table 1:** The domains of the Theoretical Domains Framework (TDF)

TDF domain	Description
Knowledge	An awareness of the existence of something
Skills	An ability or proficiency acquired through practice
Social/professional role and identity	A coherent set of behaviours and displayed personal qualities of an individual in a social or work setting
Beliefs about capabilities	Acceptance of the truth, reality, or validity about an ability, talent, or facility that a person can put to constructive use
Optimism	The confidence that things will happen for the best, or that desired goals will be attained
Beliefs about consequences	Acceptance of the truth, reality, or validity about outcomes of a behaviour in a given situation
Reinforcement	Increasing the probability of a response by arranging a dependent relationship, or contingency, between the response and a given stimulus
Intentions	A conscious decision to perform a behaviour or a resolve to act in a certain way
Goals	Mental representation of outcomes or end states that an individual wants to achieve
Memory, attention and decision processes	The ability to retain information, focus selectively on aspects of the environment, and choose between two or more alternatives
Environmental context and resources	Any circumstance of a person’s situation or environment that discourages or encourages the development of skills and abilities, independence, social competence, and adaptive behaviour
Social influences	Those interpersonal processes that can cause an individual to change their thoughts, feelings, or behaviours
Emotion	A complex reaction pattern, involving experiential, behavioural, and physiological elements, by which the individual attempts to deal with a personally significant matter or event
Behavioural regulation	Anything aimed at managing or changing objectively observed or measured actions

Note: Data is taken from Cane et al [14]

It is important that we understand what is viewed as a barrier or facilitator to the adoption, promotion and facilitation of preventive advice and treatment. This is key to improving service provision, appropriately educating dental professionals, and facilitating the effectiveness of their interactions with patients. The aim of this review is to identify the perceived barriers and facilitators to preventive oral health care from the perspectives of the oral healthcare team within the general dental practice. Barriers and facilitators will be mapped onto the TDF to understand the key psychological determinants of providing preventive oral health care.

## Methods

A scoping review was undertaken in 2015 to inform qualitative interviews which explored the barriers and facilitators of providing preventive advice in the primary dental care setting from the perspective of patients, oral healthcare professionals, dental insurers and dental policy makers, for the ADVOCATE project [15]. This review is based on this initial review but has been refined to focus only on the perspective of the oral healthcare team.

This scoping study was undertaken by following the 5-stage framework for conducting a scoping study outlined by Arksey and O’Malley [16]: Stage 1: identifying the research question, Stage 2: identifying relevant studies, Stage 3: study selection, Stage 4: charting the data and, Stage 5: collating, summarizing and reporting the results. The scoping study is reported in line with the Preferred Reporting Items for Systematic Reviews and Meta-Analyses Scoping Review extension (PRISMA-ScR) (Supplementary file 1) [17].

### Eligibility criteria

The PCC (Population, Concept, Context) framework [18] was used to develop the inclusion and exclusion criteria (Table 2).

**Table 2:** Inclusion and exclusion criteria

	Inclusion	Exclusion
Population	- Qualified dentists, dental therapists, dental hygienists, and dental nurses. - Delivery to the adult population.	- Unqualified dentists, dental therapists, dental hygienists, and dental nurses. Other non-dental professionals. - Delivery to children. - Specialised populations such as: pregnant women, geriatric patients, those with special health needs e.g., cancer, diabetes & HIV, those with mental health disorders, those with eating disorders, those with special educational needs, patients suffering from dental anxiety, homeless people, refugees, and prisoners.
Concept	- The elicitation of information on barriers and facilitators to preventive oral health care. - Prevention can include broadly preventive measures to oral hygiene or specific strategies such as the use of fluoride varnishes, floss, sealants, toothpastes (as long as prevention is explicitly expressed, and barriers and facilitators are discussed). - Any preventive advice that can be offered chair side by a trained dental professional and not requiring specialist training.	- Studies which did not address, understand, or investigate barriers and facilitators to preventive oral health care or explicitly state that an item was a barrier or facilitator. - Studies that compared the outcome of prevention over restorative care without addressing reasons for differences or addressing barriers/facilitators to prevention. - Studies which included measures such as the number/percentage of preventive approaches used or provided information about prevention without including any information on barriers/ facilitators to prevention. - Specific smoking cessation programmes, HPV (Human papillomavirus) advice, tailored diet interventions, information not routinely delivered in general dental practice. - Studies where multiple populations were included, and the findings could not be differentiated between groups.
Context	- General dental practice. - Any developed economy as defined by United Nations country classifications [19]. - Studies published after 1996. - Studies available in English.	- Any settings that are not the general dental practice such as hospital settings, care homes, academia, primary care. - Any country not defined as a developed economy by the United Nations country classification [19]. - Studies published before 1996. - Studies not available in English as there was no funding for translation.

### Search strategy

The search strategy was developed in OVID MEDLINE by an Information Specialist in conjunction with the review team. The strategy consisted of key search terms focusing on the dental team, prevention in oral health care, barriers and facilitators, attitudes, and knowledge. Key words and MESH headings were combined using the Boolean operator OR and then combined using the Boolean operator AND (Supplementary file 2).

The Medline strategy was adapted in all other databases. The following electronic databases were searched: Cochrane Central Register of Controlled trials, Cochrane Database of Systematic Reviews, Conference Proceedings Citation Index- Science, PsycInfo, EMBASE, Web of Science, MEDLINE (+ Epub and In-Process & Other Non-Indexed Citations), and SCOPUS. Searches were conducted in November 2015 to inform the ADVOCATE project and updated in June 2023. Contact with an expert in the field was also established to ensure that no relevant studies were missed. Hand searching of included studies reference lists was also undertaken against the inclusion criteria. Limits applied were papers in English and published after 1996. We only included papers published after 1996 as we felt that these more accurately reflected the modern-day dental system across countries.

### Selection of sources of evidence

Results were exported to Endnote and duplicates removed. Studies were included if they investigated barriers and or facilitators to preventive oral health care in the dental setting. The records were divided between five reviewers (KVC, HL, JC, JO, SE) so that each title and abstract was screened independently by at least two reviewers to identify potentially relevant studies against the inclusion/exclusion criteria. In case of disagreement, a consensus was reached through discussion and consultation with the remaining two/three reviewers. For those studies which met the inclusion criteria the full text of the study was reviewed by at least two of the five reviewers independently. Full text papers that did not meet the inclusion criteria at this stage were excluded.

### Data extraction

A data extraction form for the study characteristics of included studies was developed in Microsoft Excel 2002. Information relating to: authors, participant characteristics, study design, method of data collection and method of analysis were extracted. The eligible studies were divided between the reviewers (KVC, HL, JC, JO & SE) and data extraction was undertaken by one researcher and checked by a second. Where data was not available authors were contacted to obtain this.

Data was sought and included for the following variables:

A. Study identification using first author’s name and year of publication.
B. Study design.
C. Participants.
D. Type of preventive oral health care.
E. The outcomes measures/investigated.
F. The barriers that are identified to influence preventive oral health care.
G. Their corresponding TDF categorisation.
H. he facilitators that are identified to influence preventive oral health care.
I. Their corresponding TDF categorisation.
J. Who the data was extracted by (researcher initials).

To extract data on variables F-I, the results sections of each included paper were read, and content was identified and coded as a barrier or facilitator to preventive oral health care where appropriate. These items were then mapped onto the 14 TDF constructs. This mapping was done independently and spilt between five researchers so that each item was coded by at least two researchers. After initial coding, the researchers met to discuss coding and mapping of the items onto the TDF. Any discrepancies were discussed and resolved as a group.

## Results

### Sources of evidence

**Fig. 1:**
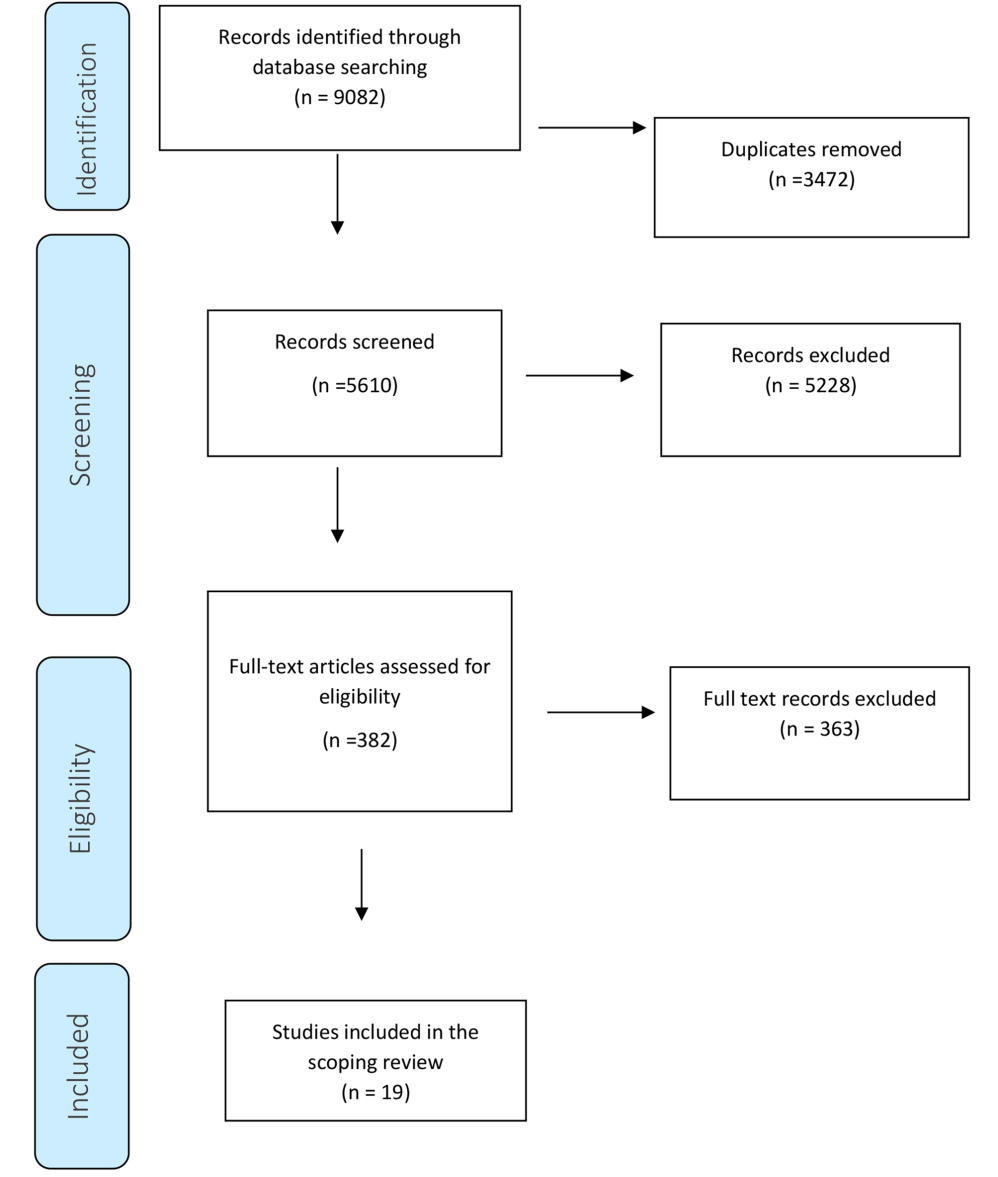
Flow diagram of study inclusion

The search strategies identified 5610 papers after deduplication which were screened. 382 full texts were assessed for eligibility and 19 were included in the final review (Fig. 1). Table 3 shows the key characteristics of each included study. Across all studies there were 7459 participants including: General dental practitioners (*n* = 5674), and dental hygienists (*n* = 1785). The most predominantly used study design was questionnaire (*n* = 15). There were a small number of studies that used qualitative methods (*n* = 3) and one cohort study (*n* = 1). The studies were conducted in nine different countries, including; USA (*n* = 7), The UK (*n* = 4), Australia (*n* = 2), Belgium (*n* = 2) Ireland (*n* = 1), Denmark (*n* = 1), Portugal (*n* = 1), Japan (*n* = 1) and Canada (*n* = 1). In terms of area of prevention that the studies focused upon, most (*n* = 9) focused on the provision of general prevention advice, two focused on fluoride application, two on fissure sealants, two on diet, one on oral health advice, one focused on oral cancer screening, one on caries prevention and one on oral hygiene instructions.

**Table 3:** Key characteristics of each study

Author and year	Country	Study design	Number of participants	Dental team member	Prevention area
Aldossri 2020	Canada	Questionnaire	932	Dentists	Oral cancer screening
Bansal 2012	USA	Questionnaire	599	Dentists	Fluoride application
Bell 2011	USA	Questionnaire	859	Dental hygienists	General prevention
Brennan 2005	Australia	Questionnaire	552	Dentists	General prevention
Catlett2016	USA	Questionnaire	360	Dental hygienists	General prevention
Dyer 2006	UK	Questionnaire and Semi-structured interviews	Questionnaire = 164 Interviews = 10	Dentists	General prevention
Fiset 2000	USA	Questionnaire	258	Dentists	Fluoride application
Kingsnorth 2020	UK & Ireland	Questionnaire	250	Dentists	Diet
Michalaki 2010	Greece	Questionnaire	977	Dentists	Fissure sealants
Rainchuso 2017	USA	Interviews	10	Dental hygienists	General prevention
Rosing 2019	Denmark	Semi-structured interviews and focus groups	8	Dentists	Oral health advice and prevention
Santos 2020	Portugal	Questionnaire	142	Dental hygienists	Dental sealants
Sbaraini 2012	Australia	Semi-structured interviews	40	Dentists	General prevention
Thevissen 2017 a	Belgium	Questionnaire	Dentists = 692 Dental hygienists = 241	Dentist, Dental hygienists	General prevention (patient motivation and oral hygiene instructions)
Thevissen 2017 b	Belgium	Questionnaire	692	Dentists	Oral hygiene instructions
Urban 2015	USA	Questionnaire	173	Dental hygienists	Caries prevention (risk assessment management)
Yokoyama 2013	Japan	Questionnaire	282	Dentists	Diet and diet counselling
Yusuf 2015	UK	Questionnaire	164	Dentists	General prevention
Kay 2003	UK	Questionnaire	15	Dentists	General prevention

### Synthesis of results – mapping barriers and facilitators onto the TDF

**Fig. 2:**
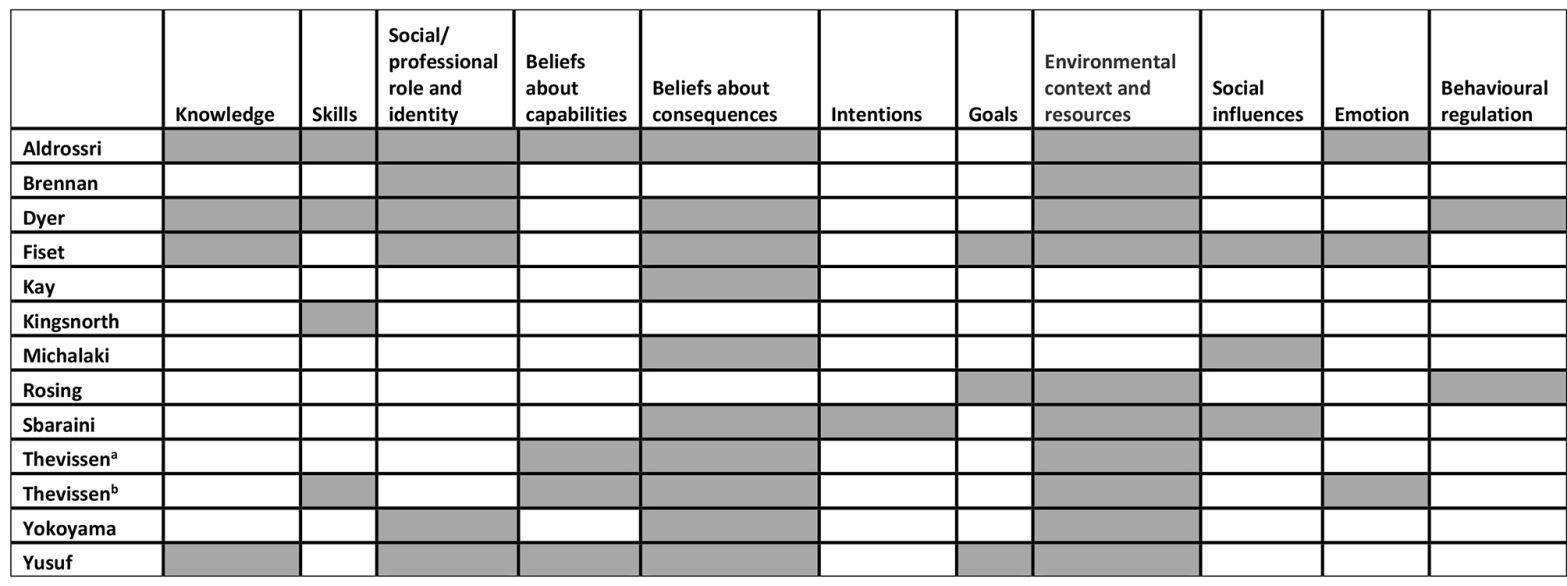
TDF domains coded per study for dentist participants

**Fig. 3:**
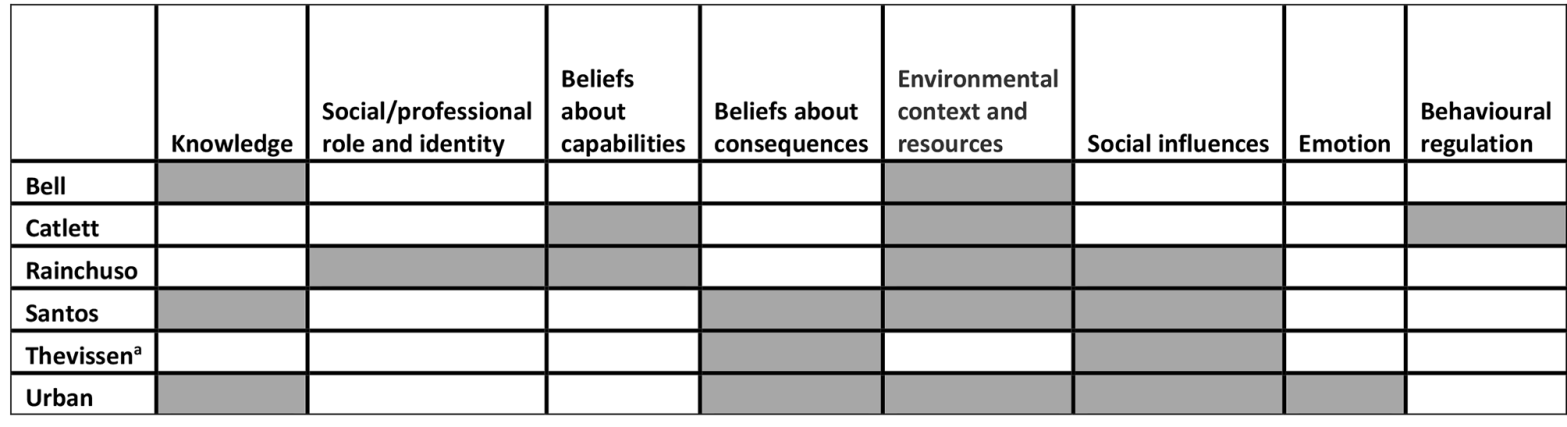
TDF domains coded per study for dental hygienist participants

Following the independent mapping of the identified barriers and facilitators onto the TDF constructs by five coders, all of the 14 constructs apart from three (Optimism, Reinforcement, and Memory, attention, and decision-making processes) were evident in the literature. It is important to acknowledge that items were allocated to domains on their best fit to ensure that we had no double coding. The data for the dentists and dental hygienists are described separately due to the differences in initial training and potential scope of practice. We have described the findings for the five TDF domains that had the greatest number of codes for the dentists and the top four for the dental hygienists. The remaining constructs are depicted in Tables 4 and 5. Figures 2 and 3 show the mapping of TDF domain per study for dentists and dental hygienists. Table 1 provides a brief description of each domain.

### Using the TDF to map barriers and facilitators to preventive oral health care for dentists

Across 13 studies focusing on dentists, there were 106 items mapped onto the TDF, of these, 48 were facilitators. The highest mapped domains for dentists were environmental context and resources, beliefs about consequences, social professional role and identity, skills, and beliefs about capabilities. These will be discussed in turn in more detail below, those not discussed are shown in Table 4.

#### Environmental context and resources

The environmental context and resources domain was identified 42 times across 10 studies [20–29]. There were 22 facilitators and 19 barriers identified within this domain. Barriers to providing preventive care existed within the practice such as a lack of remuneration or funding for providing the service [20, 22–25, 27, 29], a lack of time [20, 27, 29] or it being viewed as a poor use of time [22]. It not being seen as a priority within their working environment (e.g. the dental practice they worked in) or by policy makers were also barriers [24, 25]. Other barriers included a lack of clinical guidelines for oral cancer exams [20], unestablished systems for practicing prevention in oral healthcare in general [25], prevention being less likely when a patient attends for an emergency visit [21] and the availability of a prevention service within the practice (such as the application of fluoride varnish) [23]. Brennan also found that patients from lower socio-economic groups were less likely to receive prevention [21].

Facilitators to providing preventive advice and treatments were evident through resources being available and the environment facilitating its delivery. These included having more time available to spend with patients [20, 29] and being reimbursed for the time spent on prevention activities [20]. Having access to a variety of sources of information to learn about prevention [23], being a dentist in a solo practice [21], and working in a capital city or urban area [21, 26], were also seen as facilitators to providing prevention. In terms of the dental team, it was a facilitator to have access to another dental professional whose specific role it was to provide prevention to patients [22, 27]. Other facilitators included the availability of more efficient screening tools and less expensive screening tools for oral cancer screening [20], prevention being viewed as a priority within the dental practice, the use of a patient centred approach [26, 27], prevention being a routine part of an appointment [28] and dentists having the resources to provide information to patients through the use of leaflets and practical demonstrations [27].

#### Beliefs about consequences

The beliefs about consequences domain was identified 21 times across 10 studies [20, 22, 23, 26, 27, 29–31]. There were 3 facilitators which included a belief that early enamel caries could be ‘cured’ [30], belief that the dental team could influence effective brushing with fluoride toothpaste [30] and having positive attitudes towards prevention [29].

Barriers included a perception that efforts to motivate patients do not necessarily correlate with results obtained [26, 27], perceiving patients as not being interested in prevention or not being compliant with advice/instructions [25–27, 29], or that discussing prevention may alienate the patient [29]. A belief that routine oral cancer exams are not necessary for each patient and that oral cancer examinations cause patients too much concern [20] were barriers. Further barriers were a belief that the effectiveness of health interventions is unlikely to be effective [22], that oral hygiene alone was sufficient to reduce carries and that fissure sealants are not required [31], that diet counselling is an ineffective method for preventing carries [30] and not being convinced about the cost: benefit ratio in applying fissure sealants [23]. Some dentists also did not see prevention as a productive use of their time [25].

#### Social/professional role and identity

The social/professional role and identity domain was identified 9 times across 5 studies [20–23, 28]. Of these, 5 were barriers and 4 were facilitators. Brennan found that male dentists were less likely to provide general preventive advice [21], however, Yokoyama found that male dentists were more likely to provide diet advice [28] and Yusuf found that being a younger, female dentist was linked to more positive attitudes towards prevention [29]. Other facilitators included experiencing enthusiastic leadership and being a health-orientated dentist [22]. Feeling as though other members of the dental team have more appropriate training and time to deliver prevention [22], as well as feeling that prevention was irrelevant to dentistry [22] were additional barriers.

#### Skills

The skills domain was identified 7 times across 5 studies [20, 22, 27, 29, 32], of these 2 were barriers and 4 were facilitators. The barriers were a reported lack of training in general health promotion and prevention [22, 29]. The facilitators were valuing more training on how to perform oral cancer exams [20], seeing dietary advice as a key component of regular patient care [32], being satisfied with the training received on the links between diet and dental health [32], dentists’ skills of persuasiveness [27] and experiencing training explicitly on prevention [29].

#### Beliefs about capabilities

The beliefs about capabilities domain was identified 7 times across 4 studies [20, 26, 27, 29]. There were 5 barriers which included a lack of confidence [29], the dentist feeling uncomfortable discussing oral cancer risk factors with patients, not feeling comfortable palpating patients’ lymph nodes, and not feeling confident performing an adequate oral cancer exam [20]. Facilitators were patient’s having confidence in the dentist [26, 27].

**Table 4:** List of the items coded to the remaining TDF domains for dentists

Domain	Author	Barrier/ Facilitator	Description
Knowledge	Aldrossri	B	Not having current knowledge about oral cancer
Knowledge	Dyer	B	A lack of knowledge on health promotion
Knowledge	Fiset	B	Being unaware of the availability of fluoride varnish
Knowledge	Fiset	B	Being unaware of the cost: benefit ratio of fluoride varnish
Knowledge	Yusuf	B	Lack of knowledge on prevention
Knowledge	Yusuf	F	Improved knowledge and understanding developed through tailored training problems
Knowledge	Rosing	F	The acquisition of new knowledge on prevention
Intentions	Sbaraini	B	An opposition to change to a more preventive approach
Goals	Fiset	B	A lack of dentist motivation
Goals	Rosing	B	Feeling motivated to give chairside delivery of prevention
Goals	Yusuf	B	A lack of dentist motivation
Social Influences	Fiset	F	Asking other dentists for advice and being asked for advice
Social Influences	Fiset	F	Having colleagues who also use fluoride varnish
Social Influences	Michalaki	F	Dentists who use fissure sealants are more likely to use other fluoride regimens
Social Influences	Sbranini	F	Having a network of other dentists with the same approach/outlook on prevention
Social Influences	Sbranini	F	A team approach rather than a single dentist changing toward to a more preventive focus
Emotion	Aldrossri	B	Perception that oral cancer examinations cause patients too much concern
Emotion	Aldrossi	B	Feeling uncomfortable discussing cancer risk factors with patients
Emotion	Thevissen	B	Perception that a fear of losing teeth motivates patients
Emotion	Fiset	F	Enjoying experimenting with new procedures

#### Using the TDF to map barriers and facilitators to preventive oral health care for dental hygienists

Across 6 studies focusing on dental hygienists there were 47 items mapped onto the TDF, of these, 18 were facilitators. The highest mapped domains for dental hygienists were environmental context and resources, social influences, beliefs about consequences and knowledge. These will be discussed in turn in more detail below, those not discussed are shown in Table 5.

#### Environmental context and resources

The environmental context and resources domain was identified 18 times as a barrier and twice as a facilitator across 5 studies [33–37]. Barriers included a lack of time [33, 37], the lack of payment/reimbursement [33, 35], the impact of the loss of Medicaid benefits for patients [35]. With regards to their working environment, practice barriers, clinic rules and bureaucratic restrictions were mentioned broadly [34, 35] as well concern over legal risks [33]. Their employers lack of familiarity with caries risk assessment and management was also a barrier to delivering prevention, whereas in settings where protocols and procedures had been developed to implement a caries prevention program, this was a facilitator [37]. Cost of prevention products such as sealants was seen as a barrier [36, 37], as was the perception of patient’s acceptance of or concerns around costs for preventive services [33, 37]. In terms of location of the practice, Santos found that those working in public clinics (or both public and private clinics) had more favourable opinions on sealants compared to those who worked in private clinics only [36]. Being able to liaise with staff in public health settings to ensure follow-up dental care for patients was seen as a facilitator [35].

#### Social influences

The social influences domain was identified 7 times across 5 studies [26, 33, 35–37]. There were 4 facilitators which focused on the use of a patient-centred approach [26], the belief that fissure sealants should be promoted among dentists and dental hygienists to encourage their use [36], the importance of relationship building with dentists in the area and community integration to support the care of patients from low-income backgrounds [35]. Barriers were a lack of internal support and a lack of communication with their employer [37] as well as losing collaborative dentists from the team [35].

#### Beliefs about consequences

The beliefs about consequences domain was identified 8 times across 3 studies [26, 36, 37] as 6 barriers and 2 facilitators to delivering prevention. The dental hygienists in Urban were not convinced that caries risk assessment and management would reduce the risk of caries, and employers felt that it could reduce the profitability of restorative work [37]. Hygienist’s perceptions around patient’s’ lack of acceptance, compliance or confusion around the caries prevention program were further barriers [37] as well as a perception of patients lack of interest [26]. Dental hygienists in Santos had some concerns that the effect of pit and fissure sealants were not long lasting and they also found it difficult to justify the costs of sealants to patients [36]. Facilitators were hygienists’ perceptions of patient’s willingness to be part of the caries prevention program [37] and that their efforts to motivate patients correlated with positive outcomes for patients [26].

#### Knowledge

The domain of knowledge was identified 6 times across 3 studies [ [33, 36, 37]. Knowledge was mostly evidenced as a facilitator to providing prevention (4/6) as dental hygienists in these studies perceived themselves to have good knowledge about prevention and caries management [36, 37]. Knowledge was associated with accessing education [37] and having more years of experience [36]. A perceived lack of education regarding prevention [33] and a lack of knowledge on how to implement caries risk management [37] were barriers.

**Table 5:** List of the items coded to the remaining TDF domains for dental hygienists

Domain	Author	Barrier/ Facilitator	Description
Social/professional role and identity	Rainchuso	F	Experiencing career satisfaction
Social/professional role and identity	Rainchuso	F	Dental hygienists perceived themselves as a change agent within the communities they serve.
Social/professional role and identity	Rainchuso	F	The role of the dental hygienist improves patient access to dental care
Beliefs about capabilities	Catlett	F	Feel prepared and competent to perform preventive dental hygiene services without dentist supervision.
Behavioural regulation	Catlett	B	Working under direct supervision of a dentist
Behavioural regulation	Catlett	F	Working under more general supervision of a dentist

## Discussion

This scoping review found that the main barriers and facilitators across oral health practitioners to providing preventive oral healthcare were the environmental context and resources, skills, knowledge, beliefs about their own capabilities and the consequences of providing prevention,, their view on whether it was part of their professional role and the impact of social influences.

Environmental context and resources were the largest barrier to providing prevention for both dentists and dental hygienists and was highlighted across the majority of the included articles at least once (15/19). Working in a supportive environmental context with the provision of adequate resources, funding and time was cited as the largest facilitator to providing prevention. There were several key elements within this. One of these was remuneration; the synthesis of the data showed that a lack of appropriate remuneration or funding was seen as a significant barrier to both dentists and dental hygienists’ ability to carry out prevention effectively in practice. The effect remuneration had on the behaviour of primary care dentists has been discussed previously [38–40], however no consensus of opinion has been reached in relation to the affect remuneration has on the treatment provided. Brocklehurst and colleagues found that when the dentists were paid by the number of patients they provided care for at the practice (capitation) rather than being paid per item of activity (fee-for-service) their appointment frequency was reduced, but they were more likely to provide prevention advice [39]. However, another study, found that a capitation approach reduced clinical activity in general, including prevention [40]. Furthermore, another study found that dentists in both the UK and Ireland felt that they should be remunerated for the time taken to provide preventive care [38]. Additionally, having sufficient time to provide preventive care was highlighted as a potential issue, although interestingly, viewing prevention as a poor use of clinical time was also discussed. Previous research also found that the priority or value that was placed on prevention within the practice affected the care provided [38] and that the provision of prevention in this setting was challenging if there was insufficient time [4]. Dyer and Robinson [22] investigated the factors which influenced health promotion in practice; time and money were factors to these activities not being undertaken. Hearteningly, they found that dentists wanted to ensure health interventions were undertaken and that expansion of the team’s role was central to this. Findings suggest that having access to appropriate resources in the practice setting along with supportive tools and resources is a facilitator for providing prevention. One study highlighted that having access to effective, low-cost screening tools for oral cancer detection which were supported by appropriate training and remuneration, would improve the ability to carry out regular preventive oral cancer checks in practice [20]. The use of supportive resources, e.g., leaflets or demonstration models, was also discussed as a facilitator to preventive care in practice. This chimes with a previous study where the use of leaflets and models supported oral health conversations between health visitors and parents of young children [41, 42]. Interestingly, a lack of policy to support the delivery of prevention was not mentioned outright in any of the included articles but was implied with the issues around resources and focus placed on prevention within the practice.

Social influences were identified as an important factor on whether preventive measures were undertaken. At a practice level, a patient-centred approach with a shared understanding amongst dentists and dental hygienists of what measures should be used were a facilitator. Babiker [43] stated that ‘exceptional patient’ care could only be achieved if the team had shared values and were able to communicate clearly. Advocating the appropriate use of fissure sealants (a preventive measure) amongst dentists and dental hygienists was seen to facilitate this activity being undertaken. This suggests that agreement and a joint decision that a course of action is correct at a practice level supports the activity happening. When considering the team’s influence, dental hygienists suggested that facilitators to providing preventive advice revolved around dentists giving support to them, having good communication between the professional groups, and having dentists with a collaborative approach being retained within the team [35, 37]. Teamwork was seen as a key aspect of oral health advice being given, coupled with clear communication between team members and patients. These facets are interlinked and show establishing and maintaining the concept, and activity of ‘prevention’ as a shared goal is important.

Beliefs about consequences was an important barrier for dentists and dental hygienists alike. This coding in the TDF is one of the few that addresses not just practitioner behaviour but how the practitioners feel they might impact upon patient behaviour and as such plays an important role in motivation to carry out preventive activities or deliver preventive advice to patients. This is important, since even those who feel well trained and knowledgeable recognise that a belief in their own capabilities does not always translate to patient behaviour change (beliefs about consequences) [44]. “Enhancing patients’ oral health related behaviour is a critical component of the preventive approach” (pg.147) [45]; the authors highlight the importance of the COM-B model in helping dental practitioners to bring this about. A systematic review of interventions to enhance oral health related behaviours found that emphasising the benefits of behaviour change was an important predictor of resultant patient behaviour change [46]. Across the included studies there was quite a lot of homogeneity of reported results seen in this category which indicates that there is a large barrier to overcome for prevention to be successful; the marker of which is that it needs to influence patients’ behaviour to change. Barriers centred around practitioners’ beliefs that patients can be reluctant to change behaviour or that their delivery of prevention would be ineffective in bringing about change and have little impact on oral health due to patients’ resistance to advice and acting upon it. The few facilitators mentioned showed that with a positive attitude towards prevention from patients’, practitioners have an ability to influence behaviours. This can be viewed considering the previous work that cited blame attribution [47], whereby each group shifts responsibility to other groups. Leggett and colleagues [38] found that dentists viewed patients as not willing to take responsibility for their oral health; in contrast, patients viewed dentists as not providing preventative advice that was clear and personalised to their needs. This is congruent with a growing reference to calls for a greater application of Patient-Centred Care and its principles within the dental literature and in policy documents. However, despite the increasing prominence of the concept, more work is needed on how to translate this into dental practice with practical advice and training for dentists [48].

Our findings show that there is variability regarding whether dentists see preventive activities as part of their professional role. This was often associated with whether they felt comfortable and confident providing prevention and whether they believed other members of the dental team were better placed to provide prevention [20, 22]. Dentists who have positive attitudes towards prevention are more likely to provide advice [49]. Not all dentists view prevention as reputable or as an attractive role for dentists; it is possible that those with a positive attitude toward prevention and see its value, are more likely to view it as part of their remit. Evidence suggests dentists who qualified recently are are more likely to view preventive activities as part of their role [29]; this could be linked to the increased focus on oral health promotion and prevention in current dental education programmes. Having the appropriate skills, knowledge, and a belief about one’s capabilities is also important here [50]. The synthesis showed that most dentists felt appropriately skilled to provide prevention through training they had received [27, 29, 32]. Recent qualitative research found that most of the dental professionals interviewed felt skilled in communication, largely knowledgeable and well trained to deliver oral health education to patients [44]. In contrast to this, our synthesis showed that knowledge can still be viewed as a barrier to providing prevention, as can a belief about capabilities to provide prevention to patients. Perceptions over role and education may be contributing to dentists’ feelings of inadequacy in terms of their capabilities and knowledge. Dentists may still perceive their role primarily as diagnosing and treating dental problems rather than actively engaging in preventive care. This perception can be influenced by their education and training, which may still prioritise curative interventions over preventive strategies despite the paradigm shift towards prevention [51]. This suggests that future research needs to address dentists’ knowledge of diet and oral hygiene advice as well their confidence in its provision.

Only one study [35] provided data on whether dental hygienists saw prevention as their role. However, the three items reported relating to this were all positive, showing that the dental hygienists who participated in this study saw preventive activities as part of their professional role. This is expected since the role of a hygienist as outlined by the General Dental Practice is to “help patients maintain their oral health by preventing and treating periodontal disease and promoting good oral health practice” (pg.7) [52]. The synthesis showed that knowledge is generally seen as a facilitator for dental hygienists in providing prevention and caries management with one study also finding that hygienists feel prepared and competent to provide prevention without supervision [34]. This is not surprising since dental hygienists’ education primarily focuses on preventive care.

### Strengths and limitations

The review was conducted in line with up-to-date published guidance for conducting scoping reviews [16, 17]. To ensure a robust and systematic approach our screening and data extraction was undertaken by 5 researchers (HL, KVC, JC, JO, SE). Whilst our searches were comprehensive and were undertaken by an experienced information specialist, due to time constraints we did not search the grey literature so it is possible that some relevant studies have been missed. In line with recommendations, a quality assessment has not been undertaken on the included studies, subsequently the quality and reliability of the studies included in this synthesis may vary. As we cannot be sure of the studies’ quality and reliability it is possible that some of our findings may have limited reliability and generalisability. However, this is a considered risk with the summarising of findings within scoping reviews. Furthermore, the aim of the scoping review was to identify and map the current available evidence according to the TDF; we did not set out to undertake a qualitative exploration of the barriers and facilitators which may have led to the potential oversimplification of complex topics in its summary.

We only included general oral health prevention such as oral health promotion, fluoride application and oral cancer prevention. This approach meant that we excluded prevention within specialised areas such as individuals who have oral cancer, those who are pregnant, have diabetes or who may receive tailored advice on alcohol or tobacco consumption. We took this approach as the aim of this review was to explore barriers and facilitators to oral health preventive advice that is received by the general population within the setting of a general dental practice. The advice given in other, more specialised settings is not relevant to the general population and will likely come with their own specific barriers and facilitators. It is possible that important lessons could be learnt by exploring the delivery of oral health preventive advice in specialist settings and this is something that warrants future exploration.

A strength of our data extraction process was that we extracted the barriers and facilitators from each included paper before mapping them onto the TDF domains. This meant that we retained a high level of detail from the results within each TDF domain which enabled us to provide descriptions for each domain rather than just numbers of how many items were listed under each domain. However, we did not allow for any items to be double coded within the TDF so we may have lost some depth in the extracted data as some items did lend themselves to being coded to more than one TDF domain.

There appears to be a publication bias towards papers with dentists as a population; subsequently, a greater number of these compared to dental hygienists met the inclusion criteria of the review. We had planned to include any dental team member within the general dental practice; however, we did not find any papers that met our exclusion criteria with dental therapists or dental nurses. Due to the differences in the classification and scope of practice between dentists and dental hygienists we separated out the findings for these two groups. However, due to the aforementioned differences between dentists and hygienists, and the differences within the structure of dental systems across countries with regards to education, funding and payment, comparisons between each group and specific future recommendations are difficult.

## Conclusions

Mapping the findings onto the TDF demonstrated how the delivery of preventive activities goes beyond the interaction of the patient and dental professional; there are multiple other factors at play that influence whether prevention is delivered and the efficacy of that delivery. Currently, the focus in the oral health literature is centred around dentist’s delivery of prevention, patients’ receptivity to prevention and how to bring about behaviour change in patients. Yet, the largest barrier and facilitator for the dental professionals included in this review was the environmental context and resources available to them. There is very little focus on public health interventions for prevention looking across all parts of dental delivery; this approach is what has been identified as the biggest barrier to prevention in practice and the biggest facilitator if it can be overcome. The findings also suggest that more work is needed to ensure dental professionals are knowledgeable, well trained, up to date and confident in their abilities to deliver preventive oral health care to patients. Further research is needed to evaluate the effectiveness of interventions that aim to promote preventive oral health care in primary care settings to understand whether they address the barriers identified in this review. In terms of implications for clinical practice, dental professionals could be proactive in assessing the impact of their environment on their ability to provide prevention and exploring ways to address this within the practice. Those with low confidence in their abilities to provide prevention should be supported to improve these skills.

### Electronic supplementary material

Below is the link to the electronic supplementary material.

Supplementary Material 1

Supplementary Material 2

## Data Availability

The datasets used during the review are available from the corresponding author on reasonable request.
