# Supplementary material for: A scoping review of dental practitioners’ perspectives on perceived barriers and facilitators to preventive oral health care in general dental practice
Source: BMC Oral Health. 2024 Feb 17;24:249. doi: 10.1186/s12903-024-04022-1 (PMC10874524; doi:10.1186/s12903-024-04022-1)
Supplement: Supplementary file 1 — Supplementary Material 1 [file 12903_2024_4022_MOESM1_ESM.docx]

**Supplementary file 1. Search Strategies.**

**Ovid MEDLINE(R) ALL <1946 to June 02, 2023>**

1 exp Dentists/ or exp dental staff/ 22405

2 (dental adj5 (team or staff)).ti,kf. 623

3 (dental adj5 (professional? or worker? or practitioner?)).ti,kf. 2871

4 hygienist?.ti,kf. 2286

5 (dental adj3 assistant?).ti,kf. 1125

6 (dental adj3 nurs*).ti,kf. 377

7 (dental adj3 therapist?).ti,kf. 230

8 dentist*.ti,kf. 45385

9 or/1-8 [Dental Staff] 64483

10 ((incent* or factor* or limit* or demand* or driver* or driving* or facilitat* or threat*) adj5 (dental or dentist*)).tw. 9152

11 ((challenge* or constrain* or experience* or motiv* or influenc* or chang* or enab* or attitude* or perception* or perceive* or belief* or believe* or opinion* or view* or standpoint*) adj5 (dental or dentist*)).tw. 22314

12 (barrier* or facilitator* or behavio?r*).tw. 1742132

13 ((interview* or qualitative or theme* or survey* or questionnaire* or focus group*) adj5 (dental or dentist*)).tw. 9558

14 health knowledge, attitudes, practice/ 123555

15 "Attitude of Health Personnel"/ 129546

16 exp *Qualitative Research/ 3546

17 focus groups/ 34306

18 interviews as topic/ 66792

19 exp *Questionnaires/ 230478

20 or/10-19 [Barriers and Facilitators] 2213848

21 (dental adj7 (provider? or service? or practice?)).tw,kf. 23168

22 (oral adj7 (provider? or service? or practice?)).tw,kf. 9587

23 evidence-based dentistry/ 1318

24 general practice, dental/ 4836

25 Dental Care/ 22378

26 Oral Health/ 19137

27 "Delivery of Health Care"/ 108013

28 State Dentistry/ 2273

29 dental facilities/ or dental clinics/ or dental offices/ 6515

30 or/21-29 [Dental Care Services] 178619

31 preventive dentistry/ or exp dental prophylaxis/ or fluoridation/ or mouth protectors/ or exp oral hygiene/ 36655

32 ((prevention or preventive* or preventative*) adj5 (dental or dentist*)).tw. 7480

33 (promotion adj5 (dental or dentist* or oral*)).tw,kf. 1763

34 health promotion/ 79441

35 healthy people programs/ 1166

36 health education/ 62916

37 consumer health information/ 4247

38 health education, dental/ 6104

39 health literacy/ 8126

40 patient education as topic/ 88051

41 teach-back communication/ 53

42 (patient? adj5 (education or literacy)).tw,kf. 47208

43 (patient? adj5 guid*).tw,kf. 60848

44 guideline/ or practice guideline/ 37060

45 Guideline Adherence/ 34731

46 ((smoking or tobacco) adj2 (cessation or quit* or stop*)).tw,kf. 38373

47 Smoking cessation/ 31549

48 or/31-47 [Practising or Promoting preventative dentistry] 468047

49 9 and 20 and 30 and 48 1161

50 limit 49 to yr="2017 -Current" 328

**Embase Classic+Embase <1947 to June 02, 2023>**

1 exp *Dentist/ 13469

2 dental assistant/ 12755

3 (dental adj5 (team? or staff)).ti,kw. 625

4 (dental adj5 (professional* or worker* or practitioner*)).ti,kw. 2917

5 hygienist?.ti,kw. 2117

6 (dental adj3 assistant*).ti,kw. 1059

7 (dental adj3 nurs*).ti. 374

8 (dental adj3 therapist*).ti. 193

9 dentist*.ti,kw. 38697

10 or/1-9 59663

11 ((incent* or factor* or limit* or demand* or driver* or driving* or facilitat* or threat*) adj5 (dental or dentist*)).tw. 9870

12 ((challenge* or constrain* or experience* or motiv* or influenc* or chang* or enab* or attitude* or perception* or perceive* or belief* or believe* or opinion* or view* or standpoint*) adj5 (dental or dentist*)).tw. 23651

13 (barrier* or facilitator* or behavio?r*).tw. 2103214

14 ((interview* or qualitative or theme* or survey* or questionnaire* or focus group*) adj5 (dental or dentist*)).tw. 10732

15 attitude to health/ 126615

16 health personnel attitude/ 88357

17 attitude to change/ 635

18 dental assistant attitude/ 15

19 exp qualitative research/ 100833

20 exp interview/ 331947

21 exp observational method/ or exp questionnaire/ 845654

22 or/11-21 [Barriers and Facilitators] 3204104

23 (dental adj7 (provider? or service? or practice?)).tw,kw. 23537

24 (oral adj3 (provider? or service? or practice?)).tw,kw. 5943

25 evidence based dentistry/ 1346

26 general practice/ and (dent* or oral).mp. 7217

27 dental practice/ 1913

28 dental facility/ 3974

29 health care delivery/ 197491

30 or/23-29 [Dental care services] 234182

31 dental prevention/ 285

32 mouth hygiene/ 30432

33 caries prevention/ 744

34 exp dental prophylaxis/ 2319

35 fluoridation/ 6453

36 tooth brushing/ 13069

37 ((prevention or preventive* or preventative*) adj5 (dental or dentist*)).tw,kw. 7999

38 (promotion adj5 (dental or dentist* or oral*)).tw,kw. 1717

39 health promotion/ or exp public health campaign/ 109475

40 dental health education/ 5851

41 health literacy/ 15701

42 *patient education/ 30792

43 (patient? adj5 (education or literacy)).tw,kw. 72849

44 (patient? adj5 guid*).tw,kw. 109302

45 exp practice guideline/ 644075

46 smoking cessation/ 64855

47 ((smoking or tobacco) adj2 (cessation or quit* or stop*)).tw,kw. 50538

48 or/31-47 [Practising or promoting prevention] 1043113

49 10 and 22 and 30 and 48 1025

50 limit 49 to yr="2017 -Current" 332

**APA PsycInfo <1806 to June 02, 2023>**

1 exp Dentists/ 501

2 (dental adj5 (team? or staff)).tw,id. 99

3 (dental adj5 (professional? or worker? or practitioner?)).tw,id. 368

4 hygienist?.tw,id. 361

5 (dental adj3 (therap* or assistant? or nurs*)).tw,id. 204

6 dentist*.tw,id. 2627

7 or/1-6 3227

8 ((incent* or factor* or limit* or demand* or driver* or driving* or facilitat* or threat*) adj5 (dental or dentist*)).tw. 419

9 ((challenge* or constrain* or experience* or motiv* or influenc* or chang* or enab* or attitude* or perception* or perceive* or belief* or believe* or opinion* or view* or standpoint*) adj5 (dental or dentist*)).tw. 992

10 (barrier* or facilitator* or behavio?r*).tw. 1158921

11 ((interview* or qualitative or theme* or survey* or questionnaire* or focus group*) adj5 (dental or dentist*)).tw. 533

12 health knowledge/ or health attitudes/ 18390

13 health personnel attitudes/ 21711

14 exp qualitative research/ 19046

15 exp Questionnaires/ 23618

16 exp group discussion/ 6800

17 interviewers/ or interviewing/ or interviews/ 15575

18 behavior change/ or change strategies/ or readiness to change/ 15232

19 "Professional Personnel Attitudes & Characteristics ".cc. 41742

20 "Qualitative Study".md. 274564

21 "focus group".md. 34693

22 "interview".md. 317390

23 or/8-22 [Barriers or Facilitators to behaviour change] 1557146

24 prevention/ 34322

25 ((prevention or preventive* or preventative*) adj5 (dental or dentist*)).tw,id. 280

26 (promotion adj5 (dental or dentist* or oral*)).tw,id. 141

27 health promotion/ or exp health education/ or health literacy/ 47860

28 (patient? adj5 (education or literacy)).tw,id. 10105

29 (patient? adj5 guid*).tw,id. 5690

30 treatment guidelines/ or best practices/ 14037

31 ((smoking or tobacco) adj2 (cessation or quit* or stop*)).tw,id. 16597

32 smoking cessation/ 14334

33 or/24-32 [Practising or promoting prevention] 122439

34 (dental adj7 (provider? or service? or practice?)).tw,id. 859

35 (oral adj3 (provider? or service? or practice?)).tw,id. 546

36 (dent* office? or dent* clinic?).tw,id. 301

37 health care delivery/ 21915

38 primary health care/ 20064

39 evidence based practice/ 19793

40 or/34-39 [Dental care services] 61220

41 7 and 23 and 33 and 40 96

42 limit 41 to yr="2017 -Current" 25

**Web of Science -** Conference Proceedings Citation Index-Science (CPCI-S), Emerging Sources Citation Index (ESCI), Science Citation Index Expanded (SCI-EXPANDED)

20 #16 AND #11 AND #6 and Conference Proceedings Citation Index-Science (CPCI-S) (Web of Science Index) 1

19 #16 AND #11 AND #6 and Emerging Sources Citation Index (ESCI) (Web of Science Index) 82

18 #16 AND #11 AND #6 and Science Citation Index Expanded (SCI-EXPANDED) (Web of Science Index) 144

17 #16 AND #11 AND #6 227

16 #15 OR #14 OR #13 OR #12 153,192

15 TS=((smoking or tobacco) NEAR/2 (cessation or quit* or stop*)) 38,503

14 TS=((prevention or preventive* or preventative*) NEAR/3 (dental or dentist*)) 4,064

13 TS=(promotion NEAR/5 (dental or dentist* or oral*)) 1,348

12 TS=(patient* NEAR/5 (education or literacy or guid*)) 110,310

11 #10 OR #9 OR #8 OR #7 4,603,628

10 TS=(((interview* or qualitative or theme* or survey* or questionnaire* or "focus group*") NEAR/5 (dental or dentist*))) 8,156

9 TS=((barrier* or facilitator* or behavio$r*)) 4,579,539

8 TS= (((incent* or factor* or limit* or demand* or driver* or driving* or facilitat* or threat*) near/5 (dental or dentist*))) 8,132

7 TS=(((challenge* or constrain* or experience* or motiv* or influenc* or chang* or enab* or attitude* or perception* or perceive* or belief* or believe* or opinion* or view* or standpoint*) near/5 (dental or dentist*))) 17,850

6 #5 OR #4 OR #3 OR #2 OR #1 18,676

5 TI=(dentist*) 15,673

4 TI=(((dental near/3 therapist*) or (dental near/3 assistant*) or (dental near/3 nurs*))) 568

3 TI= ((dental near/3 hygienist*)) 492

2 TI=(((dental near/5 (professional* or worker* or practitioner*)))) 1,957

1 TI=(dental near/5 (staff or team)) 332

**Cochrane Central Register of Controlled Trials (Wiley)**

**And**

**Cochrane Database of Systematic Reviews (Wiley)**

#1 MeSH descriptor: [Dentists] explode all trees

#2 MeSH descriptor: [Dental Staff] this term only

#3 dentist*:ti,kw

#4 (dental near/5 (assistant* or nurs* or staff* or team* or therapist* or professional* or worker* or practitioner* or hygienist*)):ti,ab,kw

#5 ("oral health" near/5 (assistant* or nurs* or staff* or team* or therapist* or professional* or worker* or practitioner* or hygienist*)):ti,ab,kw

#6 {or #1-#5}

#7 ((incent* or factor* or limit* or demand* or driver* or driving* or facilitat* or threat*) near/5 (dental or dentist*)):ti,ab,kw

#8 ((challenge* or constrain* or experience* or motiv* or influenc* or chang* or enab* or attitude* or perception* or perceive* or belief* or believe* or opinion* or view* or standpoint*) near/5 (dental or dentist*)):ti,ab,kw

#9 (barrier* or facilitator* or behavio*r*):ti,ab,kw

#10 ((interview* or qualitative or theme* or survey* or questionnaire* or focus group*) near/5 (dental or dentist*)):ti,ab,kw

#11 MeSH descriptor: [Qualitative Research] explode all trees

#12 MeSH descriptor: [Attitude to Health] explode all trees

#13 MeSH descriptor: [Attitude of Health Personnel] explode all trees

#14 MeSH descriptor: [Surveys and Questionnaires] this term only

#15 MeSH descriptor: [Interviews as Topic] this term only

#16 MeSH descriptor: [Focus Groups] this term only

#17 {or #7-#16}

#18 ((prevention or preventive* or preventative*) near/5 (dental or dentist*)):ti,ab,kw

#19 (promotion near/5 (dental or dentist* or oral*)):ti,ab,kw

#20 (patient* near/5 (education or literacy or guid*)):ti,ab,kw

#21 ((smoking or tobacco) near/2 (cessation or quit* or stop*)):ti,ab,kw

#22 MeSH descriptor: [Preventive Dentistry] explode all trees

#23 MeSH descriptor: [Health Promotion] 2 tree(s) exploded

#24 MeSH descriptor: [Health Education] explode all trees

#25 MeSH descriptor: [Smoking Cessation] this term only

#26 MeSH descriptor: [Guideline] explode all trees

#27 MeSH descriptor: [Guideline Adherence] this term only

#28 {or #18-#27}

#29 #6 and #17 and #28

### **Scopus**

( TITLE ( dentist OR ( ( dental OR oral ) W/5 ( team OR staff OR professional OR worker OR practitioner OR assistant OR nurse OR therapist OR hygienist ) ) )

AND

TITLE-ABS-KEY ( ( dent* OR oral ) W/5 ( challenge OR experience OR motivate OR influence OR change OR enable OR attitude OR perception OR perceive OR belief OR believe OR opinion OR view OR standpoint OR interview OR qualitative OR survey OR questionnaire OR "focus group" ) )

AND

TITLE-ABS-KEY ( ( ( dent* OR oral ) W/5 ( prevention OR preventive OR preventative ) ) OR "health promotion" OR "patient education" OR "health literacy" OR "smoking cessation" OR "tobacco cessation" ) )
